# PP2C-Mediated ABA Signaling Pathway Underlies Exogenous Abscisic Acid-Induced Enhancement of Saline–Alkaline Tolerance in Potato (*Solanum tuberosum* L.)

**DOI:** 10.3390/plants14131921

**Published:** 2025-06-23

**Authors:** Xinhui Yang, Zisheng Liu, Jun Chen, Wangjun Zhang, Wenhui Yang, Congang Shen, Yichen Kang, Shuhao Qin

**Affiliations:** State Key Laboratory of Aridland Crop Science, College of Horticulture, Gansu Agricultural University, Lanzhou 730070, China; 13893197871@163.com (X.Y.); sagiri7210@gmail.com (Z.L.); 18597730715@163.com (J.C.); 19993691431@163.com (W.Z.); y2478626991@126.com (W.Y.); 13733170735@163.com (C.S.)

**Keywords:** saline–alkali stress, potato, ABA, PP2C, RNA interference

## Abstract

Saline–alkali stress severely inhibited potato growth, yield, and quality, and exogenous abscisic acid (ABA) played an important role in plant stress resistance. In this study, potato tissue culture seedlings were used as experimental materials, the control group was cultured in the MS medium without adding any substances, and the treatment group was cultured in MS medium supplemented with 50 mmol/L NaHCO_3_ or 50 mmol/L NaHCO_3_ + 38 µM ABA, respectively. To explore the effect of exogenous ABA on the biological characteristics of potato plants under saline–alkali stress, a genetic improvement strategy was designed based on PP2C (PGSC0003DMT400046381), a key gene of the ABA signaling pathway. The results showed that saline–alkali stress led to leaf greening, wilting, and root development stunting, while exogenous ABA treatment significantly alleviated stress damage. PP2C negatively regulates ABA signaling. SnRK2s are activated when PP2Cs are inactivated during the ABA response. Compared with wild-type CK, it was found that TG lines had increased SOD and POD activities, increased carotenoid and ABA contents, reduced the increase in Na^+^ content and the decrease in K^+^ content, and interfered with PP2C (PGSC0003DMT400046381) to significantly enhance potato salinity–alkali resistance. This study provides a theoretical basis and technical path for the analysis of ABA-mediated plant stress resistance mechanism and the breeding of potato stress resistance varieties.

## 1. Introduction

Saline–alkali stress is one of the major abiotic stresses faced by global agricultural production. Sodium carbonate (Na_2_CO_3_) and sodium bicarbonate (NaHCO_3_) in soil are among the main factors limiting agricultural production on nearly 200 million hectares of land worldwide [1]. According to the Food and Agriculture Organization of the United Nations, about 1.4 billion hectares of land worldwide, or 10% of the world’s total land area, are affected by salinization, and another 1 billion hectares are at risk of salinization due to improper irrigation, overfertilization, and climate change [2]. When the salinity content in the soil is too high, the plant absorbs a large amount of salt and alkaline substances and accumulates in the body, which increases the Na^+^/K^+^, destroys the integrity of the plant cell membrane, affects nutrient absorption, hinders photosynthesis, and can also cause ionic toxicity, high pH damage, and nutrient deficiency [3,4], which ultimately leads to reduced crop yield or even no harvest. With the intensification of the contradiction between population growth and the reduction in cultivated land resources, saline–alkali stress has become a major bottleneck threatening global food security.

Potato (*Solanum tuberosum* L.), the fourth largest food crop in the world, is a staple food source for more than 1.3 billion people, with an annual production of more than 370 million tons. However, soil salinization can seriously affect the metabolic activity of potatoes, inhibit their growth and development, reduce their photosynthetic rate, and impede protein synthesis, thus negatively affecting potato survival yield and quality [5,6]. Currently, saline–alkali stress studies on potatoes are mainly on neutral salt stress, mainly NaCI, and compared with neutral salt, the alkaline salt NaHCO_3_ in soil causes more serious harm to plant growth. Therefore, it is of strategic significance to carry out research on the mechanism of saline–alkali tolerance in potatoes and cultivate resistant varieties to guarantee food security in saline areas.

Plants coordinate abiotic stress responses through a complex network of hormone signaling. When plants perceive stress signals, they regulate a series of their metabolic activities to activate adaptive response mechanisms [7]. Phytohormones exhibit different regulatory effects at different stages of plant growth and development, in different parts of the plant, and under different external environments [8]. Four hormones, ethylene, salicylic acid, jasmonic acid, and abscisic acid (ABA), are recognized as stress-responsive hormones. Among them, ABA is considered the central regulator of “stress hormones” [9]. The expression of stress-related genes is rapidly up-regulated under various abiotic stresses [10,11]. An exogenous application of ABA triggered similar changes in the expression of stress-related genes, suggesting that environmental stress promotes the production of ABA, and that plant external environment perception and adaptive developmental responses are coordinated by endogenous ABA signaling [12]. ABA synthesis is one of the fastest responses to stress in plants [13,14]. Studies have shown that exogenous ABA treatments can significantly improve salinity tolerance in maize and other crops, and the mechanisms involved in the induction of plant salinity tolerance may be the inhibition of stomatal opening through the inhibition of K^+^ efflux channels, the up-regulation of the antioxidant defense system, the maintenance of ion homeostasis through the enhancement of selective ion uptake, improvement in root architecture through the inhibition of lateral root development, the enhancement of the K^+^/Na^+^ ratio, and osmotic regulation through the accumulation of soluble sugars and proteins [15,16,17,18]. However, its regulatory mechanism in potatoes remains unclear. According to our previous studies, ABA signaling may play a positive role in potato salinity stress response. Therefore, studying the specific regulation of ABA signaling in potato salinity response is a key breakthrough in revealing its salinity tolerance mechanism. PP2C acts as a negative regulator of ABA signaling: when PP2C is inactivated, SnRK2 kinases become activated, thereby promoting ABA signal transduction. Additionally, PP2C suppresses melatonin biosynthesis by interacting with the PMTR1 receptor and inhibiting its melatonin-binding capacity [19,20]. Therefore, investigating PP2C’s role in plant hormone biosynthesis and signaling pathways is critical.

However, the precise role and molecular mechanism of ABA signaling in regulating potato response to saline–alkali stress remain largely unexplored. To address this knowledge gap, we subjected potato plants to NaHCO_3_-supplemented MS medium with or without ABA treatment, followed by phenotypic analysis and transgenic vector construction. A comparative evaluation of transgenic versus wild-type lines under alkaline salt stress revealed key components of the ABA-regulated molecular network governing salt tolerance, providing both theoretical insights and molecular targets for stress-resistant potato breeding.

## 2. Results

### 2.1. Effects of Exogenous ABA Treatment on Growth and Development of Potato Plants Under Saline–Alkali Stress

**Figure 1:**
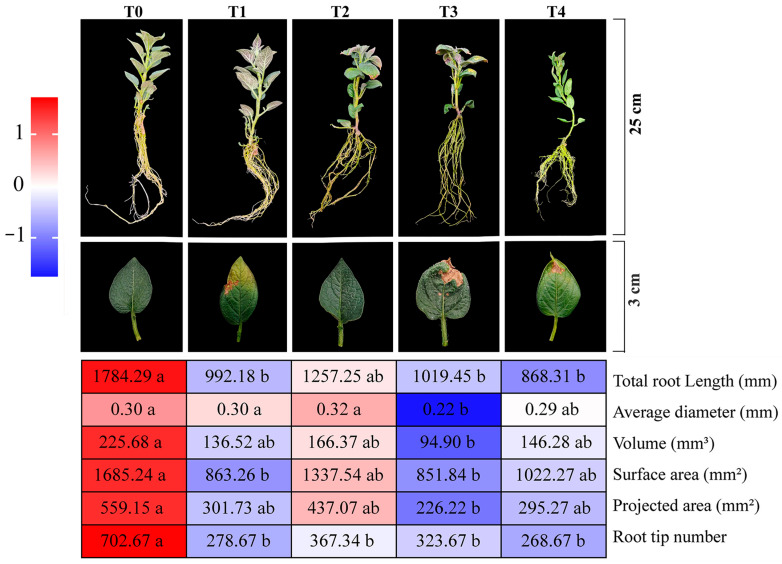
The effects of different treatments on the root phenotype and root system development of potatoes. Different lowercase letters indicate significant differences at *p* < 0.05. T0: cultured in standard MS medium without adding any treatment substances; T1: cultured in NaHCO_3_ medium for 3 days; T2: cultured in NaHCO_3_ medium and ABA-treated for 3 days; T3: cultured in NaHCO_3_ medium for 6 days; T4: cultured in NaHCO_3_ medium and ABA-treated for 6 days (the same below).

The phenotype of potato plants changed with increasing duration of NaHCO_3_ stress (3 d–6 d) (Figure 1). At 3 d of stress, T1 and T3 plants showed wilting, accompanied by the curling of leaf margins and a loss of chlorophyll, while T2 and T4 showed only slight wilting symptoms. At 6 d, T3 plants showed typical salt-damaged phenotypes: leaf chlorosis, crumpled and deformed leaves, and leaf tip necrosis, and severely inhibited root development. Although one T4 plant showed wilting and chlorosis, its root parameters were superior to those of T3. Compared to T3 plants, T4 plants exhibited a 30% increase in average root diameter and projected root area, a 54% increase in root volume, and a 20% increase in root surface area. And the quantitative morphological data showed that the root surface area (851.84 mm^2^) and root volume (94.90 mm^3^) of T3 were lower than those of the other treatments, and the growth parameters of T2 were the closest to those of the CK group.

### 2.2. The Key Pathway of ABA in Potato Response to Saline–Alkali Stress

**Figure 2:**
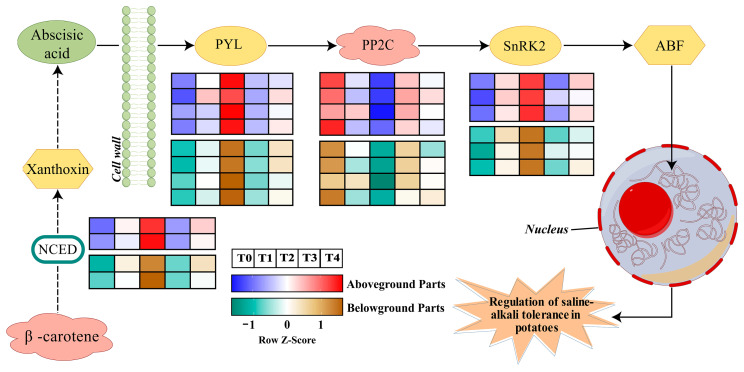
A model illustrates the changes in ABA synthesis and signal transduction pathways that are regulated by exogenous ABA in potatoes under saline–alkali stress.

To investigate the regulatory role of ABA in potatoes under saline stress, a pathway model was constructed (Figure 2) in which potato plants activate the ABA response through a multilevel regulatory network under saline stress conditions. The stress signal first promotes the accumulation of the 9-cis epoxy carotenoid dioxygenase (NCED), a key rate-limiting enzyme for ABA biosynthesis, through the activation of NCED gene family members PT0039054 (NCED) and PT0004980 (NCED), which in turn induces a significant elevation of the endogenous ABA content. The elevation of the ABA concentration triggers the PYL receptor family genes (PT0005378, PT0023511, PT0028658, PT0045156) and SnRK2 kinase gene (PT0067426, PT0060760) expression activation to form the PYL-PP2C-SnRK2 core signaling module. Notably, the expression of type 2C protein phosphatase (PP2C) genes PT0046381 (PP2C), PT0075332 (PP2C), and PT0077988 (PP2C) was significantly down-regulated in the 6 d stress treatment. Among them, the relative expression of PT0046381 was more variable, and its dynamic expression pattern was significantly negatively correlated with the degree of plant phenotypic damage. The inhibition of PP2C expression effectively lifted its constitutive inhibitory effect on SnRK2 kinase, which in turn enhanced the level of phosphorylation activation of the ABA response element binding factor (ABF).

### 2.3. Identification of Transgenic Potatoes

**Figure 3:**
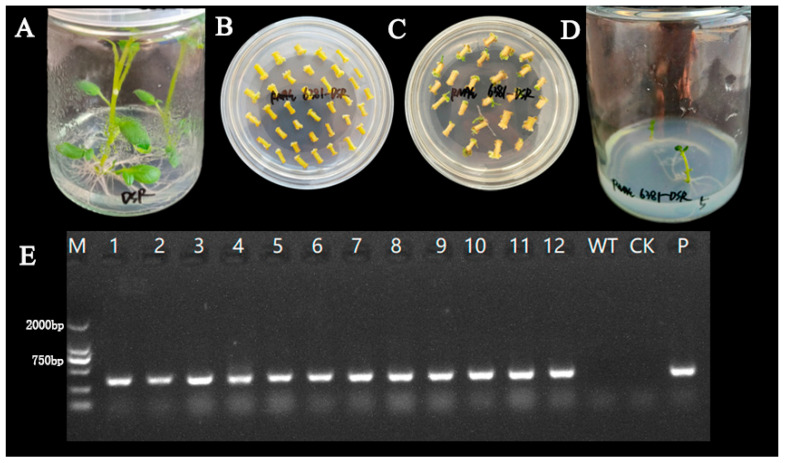
Cultivation and identification of genetically modified potatoes. Cultivation without bacterial seedlings (**A**); induction of callus tissue (**B**); generate resistant seedlings (**C**); induce the roots of resistant seedlings (**D**); positive identification of transgenic strains (**E**); M: marker DL2000; 1–12: transgenic strains; WT: non-genetically modified strain; CK: ddH_2_O; P: agrobacterium suspension.

Recombinant RNAi interference vectors were introduced into potato healing tissues by the agrobacterium GV3101-mediated method, and complete regeneration plants were obtained after resistance screening and plant regeneration culture. The genomic DNA of transgenic and control plants was extracted using agrobacterium suspension as the positive control and non-transgenic strains as the negative control for PCR identification. The results showed that 12 regenerated seedlings detected bands of the same size as those of the positive control, indicating that they were successfully transgenic (Figure 3).

### 2.4. Changes in Physiological Characteristics of Transgenic Potatoes Under Saline–Alkali Stress

**Figure 4:**
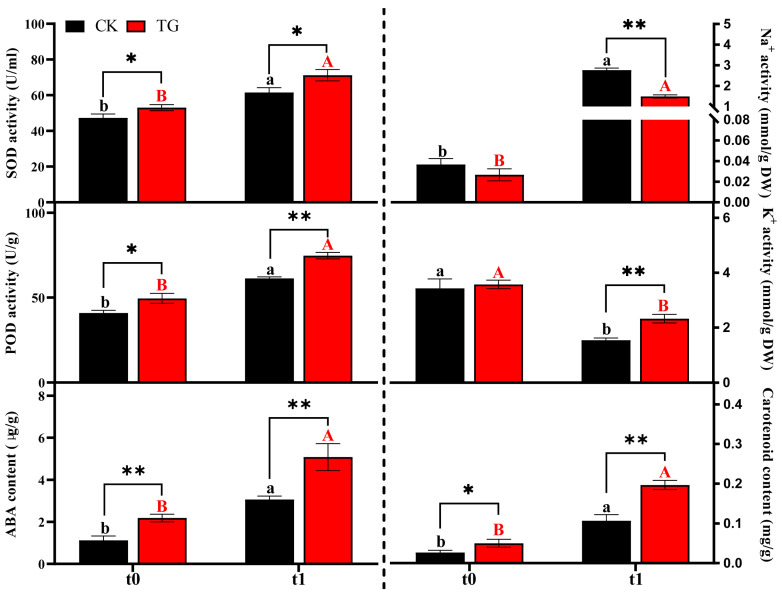
The changes in physiological characteristics of different potato strains under saline–alkali stress. Different letters and * indicate significance of *p* < 0.05 levels, ** indicate significance of *p* < 0.01 levels. Lowercase letters denote the statistical significance of differences in the CK across different stress time points. Uppercase letters denote the statistical significance of differences in the TG across different stress time points. * represents the significance of difference between different strain under the same stress time.

As shown in Figure 4, SOD activity was significantly higher under stress conditions (t1) compared with non-stress (t0); compared to t0, TG increased by 34% and CK by 30% at t1. And the activity values of the transgenic strains, TG, were significantly higher than those of the non-transgenic strains, CK, in both t0 and t1; POD activity showed a similar trend: the POD activity of the TG plants was significantly better than that of CK under both stress and non-stress conditions. The stress environment induced a significant accumulation of ABA and carotenoid content and the contents of both were significantly higher in TG plants than in CK at both t0 and t1. For ABA content, TG exceeded CK by 95% at t0 and 66% at t1. Carotenoid levels in TG were 66% and 81% higher than CK at t0 and t1, respectively. The Na^+^ contents of TG plants and CK plants increased significantly after stress, but the Na^+^ accumulation of TG plants was significantly lower than that of CK at both t0 and t1, and the increase in Na^+^ induced by stress was 1.46 mmol/g DW, which was lower than CK (2.72 mmol/g DW). Meanwhile, K^+^ content was reduced in both TG and CK plants after stress, the reduction in TG was 1.24 mmol/g DW, and the reduction in CK was 1.89 mmol/g DW, but TG plants were able to maintain higher K^+^ contents under stress.

## 3. Discussion

Salt and alkali stress causes metabolic disorders by interfering with carbon and nitrogen metabolism, hormone balance, and other physiological processes in potatoes. These negative effects act synergistically to significantly inhibit the growth and development of potatoes, resulting in a significant reduction in yield and a serious decline in quality. Abscisic acid (ABA), as a core regulatory hormone for plants to cope with abiotic stresses, plays a crucial role in potato’s resistance to saline and alkaline adversity. It has been demonstrated that the physiological functions of plant roots are significantly affected under salt-stress conditions. The surface area of plant roots was drastically reduced, which severely weakened the water uptake capacity of the root system, and then limited the process of cell division and elongation, which ultimately led to the shortening of root length and a significant reduction in root biomass [21,22]. Our experimental results showed that potato plants exogenously applied with ABA exhibited significant advantages in morphological growth indexes. Compared with the control group subjected to stress without exogenous ABA, the root length of ABA-exposed plants increased significantly, and the root system was able to extend more widely; the root surface area and root volume increased significantly, providing a larger interface for the exchange of substances between the root system and the external environment; and the root biomass was significantly elevated, which reflected a more robust root growth and enhanced the plant’s ability to survive in saline and alkaline environments. In the aboveground part, the height of potato seedlings increased and the stem thickness became thicker when ABA was applied, which provided a more solid support structure for the plants; the leaf area increased, which was conducive to improving the efficiency of photosynthesis and providing more energy for the growth of the plants; at the same time, the degree of wilting in the leaves was significantly reduced, and the normal physiological function and morphological structure of the leaves were maintained. Similar findings were verified in crops such as trifoliate orange [23] and wheat [24], further indicating that ABA has a general regulatory role in plant response to saline–alkali stress and improvement in plant growth.

In view of the key role played by ABA in mitigating abiotic stress injury in plants, its biosynthetic pathway and transporter process have become current research hotspots in the field of plant adversity biology. The biosynthetic pathway of ABA in higher plants is the C40 indirect pathway. The process begins with the polymerization of methyl erythritol phosphate (MVA) to form C40 precursor carotenoids; subsequently, also within the plastid, carotenoids are converted to zeaxanthin. Zeaxanthin is cyclized to produce epoxidized carotenoids, such as 9-cis-purple xanthin and 9-cis-neoxanthin. These epoxy carotenoids are further cleaved to produce xanthoxin (XAN). Eventually, XAN is translocated from the plastid to the cytosol, where it is converted to ABA in a series of reactions [25,26]. Carotenoids are significant for plant adversity adaptation. Studies have shown that when plants encounter environmental stresses such as intense light radiation, extremely high temperatures, and soil drought, they will activate their regulatory mechanisms to enhance their tolerance to adversity by regulating the synthesis and metabolism of carotenoids and the accumulation of substances, effectively mitigating the negative impacts of environmental stresses on the growth and development of plants, and safeguarding the maintenance of normal plant life activities [27]. Carotenoid expression enhances plant heat tolerance in Arabidopsis thaliana [28] and enhances plant resistance to salinity, drought, and oxidative stresses by increasing photosynthetic efficiency and biomass in tobacco, sweet potato, and tomato [29,30]. Brassica plants build resistance to saline, drought, and cold stresses by producing specific oxidized derivatives [31]. In the present study, we found that the carotenoid content in transgenic potato strains was higher than that in non-transgenic strains, suggesting that carotenoids play an important role in saline and alkali resistance in potatoes.

ABA signaling plays an extremely important role under abiotic stress. The stress environment often triggers osmotic stress in plants, which promotes the rapid accumulation of ABA as the core stress response hormone, and ABA regulates the expression of downstream stress response genes by integrating multiple stress signals [32]. When the concentration of ABA reaches the threshold, the ABA receptor protein PYL/PYR rapidly senses and binds to ABA molecules, and then binds to the protein phosphatase PP2C, relieving the negative regulatory effect of PP2C on SnRK2 and promoting the activation and massive expression of SnRK2 [33,34]. Activated SnRK2 further phosphorylates downstream ABA response element (ABRE)-binding proteins (AREBs) and ABRE-binding factors (ABFs), initiating a series of gene expression regulatory programs to deliver ABA signaling [35,36,37,38]. By regulating the ion balance and osmotic pressure changes in stomatal guard cells, the stomatal closure was induced to reduce water dispersion loss. At the same time, it regulates the expression of genes related to seed development and maintains seed dormancy, thereby helping plants resist the damage caused by stress [39]. Phenotypically, this conferred enhanced stress tolerance, manifesting as reduced morphological damage—including less wilting, shriveling, and necrosis—under stress conditions. Therefore, we believe that a breakthrough in potato saline–alkali resistance can be achieved by studying the ABA signaling pathway.

PP2C, a branch of protein phosphatase, can catalyze the dephosphorylation of proteins and other biological molecules, and plays an important role in cell signaling networks by removing phosphate groups to alter the activity state of substrates [40,41,42]. PP2C is involved in many aspects of plant physiology, not only regulating basic growth and development processes, such as cell division and tissue differentiation, but also being deeply involved in important signaling pathways such as ABA signaling and plant immune defense [43,44,45]. It has been found that the PP2C protein family is an important negative regulator of the ABA signaling pathway [46,47], which is involved in ABA-mediated plant stress resistance signaling and plays an important regulatory role in plants in response to abiotic stresses such as drought, high salt, and low temperature [48]. Under drought stress, the overexpression of the ZmPP2C-A10 gene in maize plants was worse than that of wild-type maize plants, indicating that the resistance of transgenic maize plants was weakened, and the ZmPP2C-A10 gene decreased the tolerance of maize plants to stress [49]. ZmPP2C-A2 and ZmPP2C-A6 transgenic Arabidopsis thaliana plants had high leaf water loss and low survival rates under stress, indicating that the ZmPP2C-A2 and ZmPP2C-A6 genes played an inhibitory role in the stress response [50]. The growth of MdPP2C24/37 transgenic Arabidopsis thaliana plants was inhibited, and chlorophyll levels decreased under stress, indicating that the MdPP2C24/37 gene had a negative regulatory effect on stress resistance [51]. Therefore, a reduction in the PP2C gene expression level can regulate the overall stress response of plants through the signaling network, significantly improve the tolerance of plants to stress, regulate physiological and metabolic processes more efficiently, maintain growth and development, and achieve survival and growth under adversity. Therefore, in this study, we interfered with PP2C through RNAi technology and found that the transgenic potato strains were more resistant to salinity compared with the non-transgenic strains, and the interference with PP2C (PT0046381) significantly alleviated the damage of potato plants under saline–alkali stress. This study demonstrates that manipulating the ABA signaling pathway, specifically through suppressing the negative regulator PP2C via RNAi, significantly enhances potato tolerance to saline–alkali stress. Next, we can investigate the expression differences in the PP2C gene family in different potato varieties and screen natural variation sites to guide molecular marker-assisted breeding.

## 4. Materials and Methods

### 4.1. Plant Material and Growing Conditions

Potato histoculture seedlings were used as experimental materials in this experiment. The variety was Solanum tuberosum cv. Desiree (laboratory-preserved germplasm). The seedlings were cultured in MS medium and placed in an incubator (the MS medium was prepared using Murashige and Skoog Basal Medium provided by Sigma-Aldrich, (Shanghai, China) with additional sucrose added). They were subjected to a 16 h daily photoperiod at 21 °C, with the ambient relative humidity maintained between 50% and 60%. LED lighting was used, with a photoperiod intensity of 200 μmol·m^−2^·s^−1^ and an illuminance level of 3000 Lx. Following 21 days of growth, the seedlings underwent treatment.

### 4.2. Experimental Design

#### 4.2.1. Experimental Design of ABA for Alleviating Alkaline Salt Stress

After 21 days of basal culture, the plant tissue culture seedlings were divided into five groups according to the differences in treatment conditions. (1) T0: This group continued to be cultured in standard MS medium without any treatment substances added. (2) T1 (NaHCO_3_ medium): This group was transferred to MS medium containing 50 mmol/L NaHCO_3_ for 3 days. (3) T2 (ABA-treated): Based on T1 treatment, 38 µM ABA was applied simultaneously for a total of 3 days. (4) T3 (NaHCO_3_ medium): This group was transferred to MS medium containing 50 mmol/L MS medium for NaHCO_3_, in a continuous treatment for 6 days. (5) T4 (ABA-treated): Based on T3 treatment, 38 µM ABA was applied synchronously for a total of 6 days. Three biological replicates were set up for each group.

#### 4.2.2. Identification of Resistance of Transgenic Plants

Transgenic strains (TG) and non-transgenic strains (CK) were placed in MS medium containing 50 mmol/L NaHCO_3_ (pH = 8.2) to simulate saline and alkaline stress, and the samples were taken before the treatment (0 d) and recorded as t0, and then continuously treated for 6 d (t1) and sampled again after the treatment, and physiological indexes were measured.

### 4.3. Determination of Morphological Indicators

Systematic phenotyping of plants in the five treatment groups was carried out, focusing on the changes in leaves, the root system, and the overall growth status. For leaves, color changes (degree of yellowing/greening) and wilting and curling patterns were recorded, and parameters such as total root length, average root diameter, and root volume were quantified using the GXY-A (Model: ScanMaker i800 Plus provided by Zhejiang topu yunnong Technology Co., Ltd. (Zhejiang, China); Website address: www.tpyn.net) root phenotyping system.

### 4.4. qRT-PCR Analysis of Key Genes

Thirteen key genes were selected for real-time fluorescence quantitative PCR (qRT-PCR) validation based on our previous study [52]. We used a gene amplification instrument provided by Hangzhou Borui Technology Co., Ltd. (Hangzhou, China), model TC-96/G/H(b)B. The conditions for qPCR are shown in Table 1. Reverse transcription of mRNA was performed using the FastKing gDNA Dispelling RT SuperMix kit provided by Tiangen Biochemical Technology Co., Ltd. (Beijing, China). Using Actin as an internal reference gene, we detected the cDNAs obtained on the fluorescent qPCR instrument using the TB Green Premix Ex Taq II kit according to the manufacturer’s instructions. The relative expression of each tested gene was calculated according to the 2^−ΔΔCt^ method [53]. Three biological replicates were set up for each set of experiments, and three technical replicates were performed for each reaction. The primers used for qRT-PCR experiments are listed in Table 2.

### 4.5. Generation of Transgenic Plants

Based on the results of the pre-screening (Section 2.1 and Section 2.2), the PT0046381 gene in the PP2C family was selected for functional validation. Using potato histoculture seedling cDNA as a template, PCR amplification was used to obtain the forward interfering fragment and reverse interfering fragment of this gene. We constructed the forward sequence and the reverse sequence to the interference carrier, and the sequence accuracy was verified by sequencing. The recombinant plasmid was transformed into agrobacterium tumefaciens GV3101 receptor cells, followed by colony PCR verification, and the positive transformants were screened and used for subsequent genetic transformation experiments.

The cut explants were placed in resuspended agrobacterium suspension, and subsequently transferred to MS co-culture medium and incubated in dark culture at 25 °C for 2–4 d. After co-culturing, the explants were transferred to an MS medium containing resistance to induce germination. The differentiated resistant buds were inoculated on resistant 1/2 MS medium for seedling strengthening and were allowed to form complete plantlets. Genomic DNA was extracted after the formation of plants and PCR-positive tests were performed.

### 4.6. Measurement of Physiological Index

Sodium and potassium ion content was measured by flame spectrophotometry, ABA content was measured by high-performance liquid chromatography (HPLC), the mobile phase was methanol and of 0.1%, phosphoric acid (9:1) was used, the flow rate was 0.8 mL/min, the injection volume was 10 μL, and the column temperature was 30 °C. Each sample was measured repeatedly three times. The hormone content was calculated through the standard curve. Superoxide dismutase (SOD) activity, peroxidase (POD) activity, and carotenoid content was measured by UV spectrophotometry using the kits BC0090, BC0170, and BC4330 for determination. The SOD, POD, and carotenoid kits were provided by Beijing Solarbio Science & Technology Co., Ltd. (Beijing, China). Determination methods for SOD and POD: Extract 0.1 g of the tissue, add 1 mL of the extract, and perform ice bath homogenization. Centrifuge 8000× *g* at 4 °C for 10 min, and take the supernatant and place it on ice for testing. Detect the OD value. Calculate according to the instructions of the reagent kit.

### 4.7. Data Analysis

Microsoft Excel 2019 software was used to count the data, SPSS 27.0 software was used to analyze the data, and Origin 2022 software was used for graphing. The data were analyzed for variance and significance using LSD ANOVA and Duncan’s new complex polarity method with a significant level of 0.05.

## 5. Conclusions

Saline–alkali stress significantly inhibited the growth of potato plants by disrupting ionic homeostasis and inducing oxidative damage, which was manifested as leaf wilting, a decrease in plant height, stem thickness, root length, and root biomass, and the inhibition of normal growth and development, while the exogenous ABA-exerted potato plants suffered from a significant reduction in stress damage, and the various morphology and growth indexes were moderated. Mechanistic studies showed that ABA promotes the expression of stress-resistant genes and enhances antioxidant defense through activation of the PYL-PP2C-SnRK2 signaling pathway, in which the inhibition of PP2C expression plays a key role in relieving the inhibition of SnRK2 activity. The transgenic plant TG synergistically regulated ABA signaling intensity, redox balance, and ion homeostasis. Under saline and alkaline stress, the transgenic plant TG showed significantly enhanced stress tolerance with increased SOD and POD activities, increased carotenoid and ABA contents, and decreased sodium–potassium ion ratio. This study reveals the central role of the PP2C-mediated ABA signaling regulatory network in the response to salinity stress in potatoes, which provides a new target for the selection and breeding of stress-tolerant varieties.

## Figures and Tables

**Table 1:** qPCR reaction conditions.

	Temperature	Time	Cycles
Step 1	95 °C	30 s	1
Step 2	95 °C	5 s	50
	60 °C	30 s	

**Table 2:** Gene IDs and primer sequences for the genes used for qPCR verification.

Gene ID	Forward Primer	Reverse Primer
PGSC0003DMT400067426	ATGGAGGAAAAGTATGAGCTTTTGA	TCAGACATAAACAGCAAAGTCA
PGSC0003DMT400060760	ATGGAAAGATATGAAATTCAGAAAGAC	TCATACCATTGAATGACAGTAACTCAC
PGSC0003DMT400060264	ATGCAGAATTACGAAGTTGTGAAGG	CGAGGTGCTGGACTTCCATC
PGSC0003DMT400046381	ATGACAGTTGCTGATTGCCA	CTACGTTTTCTTCTTGAATTTCCTCTG
PGSC0003DMT400075332	ATGGAGGAGATGTATATGGTTGCA	TTAGCTAGGAGAAAACATACCGA
PGSC0003DMT400077988	ATGGCAGAGGTCTGTTTTGGA	TTAACGTACATGAGAGCAGCCA
PGSC0003DMT400046381	ATGACAGTTGCTGATTGCCA	CTACGTTTTCTTCTTGAATTTCCTCTG
PGSC0003DMT400005378	ATGGAGCAATCCGATAACTCA	TTAGGAGATCTCACCGTTACCAC
PGSC0003DMT400023511	ATGAACGCTAATGGATTCTGCG	TTAGACCTGATCAATGGGTTCTGT
PGSC0003DMT400028658	ATGCCTCCCAGTTCTTCAGA	TCATCTGCTTGAATTCCGTGC
PGSC0003DMT400045156	ATGGATAGTAAACCGGAAACGTCA	TCACCTGTGACTTACATCACT
PGSC0003DMT400039054	ATGACTTCCACAATTGCAAA	TTATACTTGATTTTGCAAGTCCT
PGSC0003DMT400004980	ATGCCGAAAGTAATAGGGATAGCA	TTATAGTTTCATAAGATCATTTTCCGT

## Data Availability

The datasets generated and analyzed during the current study are available from the NCBI repository, https://www.ncbi.nlm.nih.gov/sra/?term=SRP298983 (accessed on 21 January 2025), and the accession number is SRP298983. The datasets used and analyzed in the current study are available from the corresponding author on reasonable request.

## Abbreviations

The following abbreviations are used in this manuscript:

ABA	Abscisic acid
PP2C	Protein phosphortase 2C
PT	PGSC0003DMT40
SnRK2	Sucrose non-fermenting-1-related protein kinase 2
SOD	Superoxide dismutase
POD	Peroxidase
qRT-PCR	Quantitative real-time polymerase chain reaction
cDNA	Complementary DNA
HPLC	High-performance liquid chromatography
NCED	9-cis epoxy carotenoid dioxygenase
PYL	Pyrabatin resistance 1-like proteins
ABF	Abscisic acid-responsive element binding factors
MVA	Methyl erythritol phosphate
XAN	Xanthoxin
PYR	Pyrabatin resistance
ABRE	Abscisic acid responsive element
